# Supplementary material for: Long COVID and Its Impacts: A Case–Control Study in Brazil
Source: Biomedicines. 2025 Jul 1;13(7):1615. doi: 10.3390/biomedicines13071615 (PMC12292682; doi:10.3390/biomedicines13071615)
Supplement: Supplementary file 1 [file biomedicines-13-01615-s001.zip › biomedicines-3683034-supplementary.pdf]

Informed Consent Form (ICF)

You are being invited to participate as a volunteer in the research entitled "MonitoraCovid: A Longitudinal Monitoring Study". The objective of this study is to compare the manifestations of Long Covid among patients who were tested using the MonitoraCovid system.
The information provided will be completely confidential and will be under the care of the research coordinating professor at the university. The only risk inherent to participating in the research is the discomfort when responding to the questionnaire. However, these are quick and general questions, which take up to 10 minutes to answer.
By agreeing to the terms of this research, you declare awareness of the confidentiality of the data, as well as its use for academic purposes only. Furthermore, your right is guaranteed to: (1) withdraw consent and participation at any time, if you deem it necessary; and (2) security of non-disclosure of personal data that could identify you.
This research is also in accordance with Brazilian Resolution No. 210, of April 7, 2016, of the National Health Council, CNS Resolution No. 466/12 and 510/16, relating to research on human beings in Brazil.
Suppose you have any questions related to any ethical aspect of this study or about the protection of the rights of the object of study. In that case, you can contact the research coordination (xxx) or the Research Ethics Committee (xxx).
After completing the questionnaire, you will automatically receive the TCLE in your email, if you wish.
The search begins next.

1. Do you agree to participate in this research?
- No
- Yes

Follow-up depends on the response.

If you answer NO to question 1:
1.1. Could you tell us why you do not agree to participate in this research?
- I am not interested in participating
- I don't feel comfortable answering the questionnaire
- It takes a long time
- I prefer not to respond
- Other (specify):____________________

Ends with thanks.

If you answer YES to question 1:

Sociodemographic data

We will send you the Informed Consent Form (ICF). Please enter the same email address registered with Monitora COVID below, that is, the email address where you received the questionnaire: We will

send you the ICF. Please enter the same email address registered with MonitoraCOVID below, that is, the email address where you received the questionnaire:

3. Email address: ________________________

4. How old are you? ________________________

5. What is your sex?
- Feminine
- Masculine
- I prefer not to respond

6. What is your education?
- No education or incomplete primary education
- Complete primary education or incomplete secondary education
- Completed secondary education or incomplete higher education
- Completed higher education or incomplete postgraduate studies
- Complete postgraduate degree
- I don't know how to answer
- I prefer not to respond

7. How do you define your skin color/race?
- White
- Black
- Brown
- Asian
- Indigenous
- I don't know how to answer
- I prefer not to respond

8. Do you have any private health insurance?
- Yes
- No
- I prefer not to respond

Clinical conditions

In this section, we will ask about your current medical conditions.

9. Do you have any of these health conditions? (you can select more than one option if necessary)
- No pre-existing medical conditions
- Heart disease
- Diabetes
- Chronic lung disease
- Immunosuppression

- Arterial hypertension
- Chronic kidney disease
- Chronic liver disease
- Asthma
- I prefer not to respond
- I don't know how to answer
- Other pre-existing medical conditions: ________________________

10. Have you been using any medication continuously lately?

- No
- Yes
- I prefer not to respond

11. What medication(s) are you currently taking? ________________________

12. Have you been vaccinated against COVID-19?

- Yes
- No
- I prefer not to respond

13. How is your vaccination schedule for COVID-19?

	Starting doses	Booster doses	Bivalent vaccine
COVID-19 Vaccine			

14. Have you ever had COVID-19 confirmed by a diagnostic test (consider a PCR test carried out by the MonitoraCovid program)?

- Yes
- No
- I don't know how to answer

15. How many times have you had COVID-19 confirmed with a positive test?

- 1
- 2
- 3
- 4
- 5

16. What was the approximate date of the last Covid diagnosis?
Day/month/year
Data: ________________________

17. Were you hospitalized when you last had COVID?

- No
- No, and I didn't need hospitalization the other times I had Covid
- No, but I needed intensive care the other times I had Covid
- No, but the other times I had Covid I needed hospitalization without intensive care (ICU or CTI)
- Yes, I needed intensive care (ICU or CTI) during hospitalisation during the last COVID diagnosis
- Yes, but I did not need intensive care (ICU or CTI) during my hospitalisation during the last COVID diagnosis
- I prefer not to respond

18. Have you ever suspected that you were infected with COVID, but did not take a confirmatory test?
- Yes
- No
- I don't know how to answer

Follow-up depends on the response.
If you answer YES to question 18:
18.1. YES answer for Covid diagnosis.
18.1.1 Health Assessment
The next questions refer to clinical manifestations possibly associated with COVID-19 and which remain to this day.
“Now” refers to how you feel right now/this week (last 7 days).
"Pre-COVID" refers to how you were feeling before you got the disease (if you got it more than once, consider it the last one).
If you can't remember an answer, just say 'I don't know'.
Rate the severity of each problem on a scale of 0 to 3, as follows:
0 = None; no problem.
1 = Mild problem; does not affect daily life.
2 = Moderate problem; affects daily life to some extent.
3 = Serious problem; affects all aspects of daily life.
19. Do you feel or have you felt short of breath?

	Now	Pre-COVID
When I'm at rest		
When I am changing position, for example from lying down to sitting		
When I'm getting dressed		

When I'm climbing stairs		

18.1.2. Do you have or have you had coughing or voice change problems?

	Now	Pre-COVID
Cough/Throat tenderness		
Voice change		

18.1.3. Do you feel or have you felt fatigue (tiredness not recovered by rest and rest)?

	Now	Pre-COVID
Fatigue levels in your usual activities		

18.1.4. Do you have or have you had changes in your sense of smell and/or taste?

	Now	Pre-COVID
Altered sense of smell		
Altered taste		

18.1.5. Do you feel or have you felt pain and/or discomfort?

	Now	Pre-COVID
Chest pain		
Joint pain		
Muscle pain		
Headache		
Abdominal pain (belly pain)		

18.1.6. Do you have or have you had any cognitive impairment?

	Now	Pre-COVID

Concentration problems		
Memory problems		
Problems with planning		

18.1.7. Do you have or have you had palpitations (racing heart) and/or dizziness?

	Now	Pre-COVID
Palpitations in certain positions, activities or at rest		
Dizziness in certain positions, activities or at rest		

18.1.8. Do you feel or have you felt any post-exercise discomfort?

	Now	Pre-COVID
Relapse hours or days after performing physical, cognitive or emotional effort		

18.1.9. Do you have or have you had problems related to anxiety and mood?

	Now	Pre-COVID
I feel anxious		
I feel depressed		
I have unwanted memories of my illness or time in the hospital		
I have unpleasant dreams about my illness or time in the hospital		
I try to avoid thoughts or feelings about my illness or time in the hospital		

18.1.10. Do you have or have you had problems with your sleep?

	Now	Pre-COVID
Difficulty falling asleep, staying asleep, or sleeping too much		

18.1.11. Do you have or have you had communication problems?

	Now	Pre-COVID
Difficulty finding words/difficulty understanding others		

18.1.12. Do you have or have you had problems walking or moving?

	Now	Pre-COVID
Difficulties when walking or moving		

18.1.13. Do you have or have you had problems with other activities of daily living?

	Now	Pre-COVID
Difficulty when carrying out broad activities, such as housework, leisure/sports activities, work, study or shopping		

18.1.14. Do you have or have you had socialization problems?

	Now	Pre-COVID
Problems when socializing/interacting with friends or caring for dependents*		

*Related to your illness and not due to your own social distancing/lockdown measures.

Health Perception
This section aims to understand how you consider your health currently and before COVID-19.

18.1.15. Imagine a thermometer where 0 represents the worst imaginable state of health (death) and 100 represents the best possible state of health. How would you rate your health today?

0 → 100 _______________________

18.1.16. Health in general, before COVID-19. Imagine the same thermometer. How would you rate your health before Covid?

0 → 100 _______________________

Go to question 19

If you answer NO to question 18:
18.2. Answer NO for Covid diagnosis.
18.2.1. Health Assessment
The next questions aim to perform a general assessment of your current health. Please answer the questions below as accurately as possible. If you can't remember an answer, just say 'I don't know'.
Rate the severity of each problem on a scale of 0 to 3:
0 = None; no problem.
1 = Mild problem; does not affect daily life.
2 = Moderate problem; affects daily life to some extent.
3 = Serious problem; affects all aspects of daily life.

18.2.2. Do you have problems with shortness of breath?

	Now
When I'm at rest	
When I am changing position, for example, from lying down to sitting and vice versa	
When I'm getting dressed	
When I'm climbing stairs	

18.2.3. Do you have problems with coughing, throat sensitivity or voice changes?

	Now
Cough/Throat tenderness	

Voice change	

18.2.4. Do you have problems with fatigue (tiredness not recovered by rest and rest)?

	Now
Fatigue levels in your usual activities	

18.2.5. Do you have problems with changes in smell and taste?

	Now
Altered sense of smell	
Altered taste	

18.2.6. Do you have problems with pain and discomfort?

	Now
Chest pain	
Joint pain	
Muscle pain	
Headache	
Abdominal pain (belly pain)	

18.2.7. Do you have problems with cognitive impairment?

	Now
Concentration problems	
Problems with memory	
Problems with planning	

18.2.8. Do you have problems with palpitations and dizziness?

	Now
Palpitations in certain positions, activities or at rest	
Dizziness in certain positions, activities or at rest	

18.2.9. Do you feel any post-exertion discomfort?
Post-exertional malaise refers to the worsening of symptoms (relapse) after minor physical or mental exertion, with symptoms typically worsening 12 to 48 hours after activity and persisting for days or even weeks.

	Now
Relapse hours or days after performing physical, cognitive or emotional effort	

18.2.10. Do you have problems related to anxiety and mood?

	Now
I feel anxious	
I feel depressed	
I have unwanted memories	
I have unpleasant dreams	
I have negative thoughts or feelings	

18.2.11. Do you have problems with your sleep?

	Now
Sleep problems, such as difficulty falling asleep, staying asleep, or sleeping too much	

18.2.12. Do you have communication problems?

	Now

Difficulty communicating/difficulty finding words/difficulty understanding others	

18.2.13. Do you have problems walking or moving around?

	Now
Difficulties when walking or moving	

18.2.14. Do you have problems with other activities of daily living?

	Now
Difficulties when walking or moving	

18.2.15. Do you have socialization problems?

	Now
Problems when socializing/interacting with friends or caring for dependents*	

*Related to your health condition and not due to your own social distancing/lockdown measures.

Health Perception
This section aims to understand how you currently view your health.

18.2.16. Imagine a thermometer where 0 represents the worst imaginable state of health (death) and 100 represents the best possible state of health. How would you rate your health today?

0 → 100 ________________________

Other symptoms
19. We're almost done. Select which of the following symptoms you have experienced in the last 6 months. Select more than one option, if necessary.

- No symptoms
- Rash (redness or changes in the skin, such as lumps and swelling)
- Skin discoloration
- New allergies, such as medicines, foods, etc.
- Excessive hair loss
- Feeling of numbness, tingling, or itching in the skin or nerve pain
- Dry eyes/eye redness
- Swelling of feet/swelling of hands

- Easy and sudden bruising/bleeding
- Changes in vision
- Difficulty swallowing solids
- Difficulty swallowing liquids
- Falls/balance problems
- Weakness, difficulty in movement and problems in limb coordination
- Ringing in the ear
- Nausea
- Dry mouth/mouth sores
- Heartburn and acid reflux
- Change in appetite
- Unintentional weight loss
- Unintentional weight gain
- Urinary urgency or incontinence (involuntary loss of urine)
- Constipation, bowel incontinence or diarrhoea
- Changes in the menstrual cycle/flow
- Waking up at night short of breath
- Thoughts about getting hurt
- Fever
- Other symptoms (specify) ________________________

20. In addition to the physical symptoms mentioned in the previous question, have you also experienced emotional and/or psychological changes in the last 6 months?

- No emotional or psychological symptoms
- Depression
- Post-traumatic stress
- Disturbance where I am
- Anxiety
- Other emotional or psychological symptoms________________________

Life habits

Here we will ask you some questions about habits and customs. We reinforce data confidentiality.

21. In the last 6 months, have you practised any type of physical exercise or sport?

- No
- Yes
- I prefer not to respond

22. What is your frequency of physical activities?

- Up to 2 and a half hours per week
- From 2 and a half hours to 5 hours per week
- From 5 hours to 10 hours per week
- More than 10 hours per week
- I prefer not to respond

23. Do you consider your diet to be healthy?

- Yes
- No
- I don't know
- I prefer not to respond

24. Which of the following best describes how often you consume fresh, unprocessed foods?
- Never
- Rarely
- A few times a week
- Daily
- Other:_______________________

25. In the last 6 months, how often have you consumed any alcoholic beverages?
- I didn't drink alcohol
- Consume 1 or 2 days a week
- Consume 3 or 4 days a week
- Consume 5 or 6 times a week
- I consumed it every day of the week
- On average 1 day per week
- On average 1 day per month
- I don't know how to answer
- I prefer not to respond

26. On occasions when you drink, how many shots, glasses or bottles do you usually have?
Consider:
BEER: 1 glass (350ml) or 1 can – 1 “DOSE” / 1 bottle – 2 “DOSES”.
WINE: 1 glass (250ml) – 2 “DOSES” / 1 bottle – 8 “DOSES”.
CACHAÇA, VODKA, WHISKEY or BRANDY: 1 “hammer” (60ml) – 2 “DOSES” / 1 “hammer” (100ml) – 3 “DOSES” / 1 bottle – more than 20 “DOSES”.
WHISKEY, RUM, LIQUOR, etc.: 1 “dose dose” (45-50ml) – 1 “DOSE”.

- 1 or 2 “doses”
- 3 or 4 “doses”
- 5 or 6 “doses”
- 7 a 9 “doses”
- 10 or more “doses”
- I don't consume alcoholic beverages

27. In the last 6 months, have you smoked?
- No
- Yes, but not daily
- Yes, daily
- I prefer not to respond

28. In the last 6 months, have you used any illicit drugs?

- No
- Marijuana/hashish/skank
- Cocaine
- Crack/oxy/merla
- Solvents (perfume launcher or "loló")
- Ecstasy/MDMA
- Heroin and/or injectable drugs
- LSD
- I prefer not to respond
- Other (specify):________________________

Thanks

We greatly appreciate your participation. Your answers will be key to identifying persistent Covid symptoms. We emphasize that the information provided is completely confidential.
